# Diagnostic performance of nucleic acid tests in tuberculous pleurisy

**DOI:** 10.1186/s12879-020-04974-z

**Published:** 2020-03-24

**Authors:** Min Han, Heping Xiao, Liping Yan

**Affiliations:** 1 Department of Clinical Laboratory, Shanghai Pulmonary Hospital, Tongji University School of Medicine, Shanghai, China; 2 Department of Tuberculosis, Shanghai Pulmonary Hospital, Tongji University School of Medicine, No. 507 Zhengmin Road, Shanghai, 200433 China

**Keywords:** Xpert MTB/RIF, AmpSure simultaneous amplification and testing, Loop-mediated isothermal amplification, Diagnosis, Tuberculosis

## Abstract

**Background:**

Tuberculous pleurisy (TBP) is the most common form of extrapulmonary tuberculosis (TB). However, rapid diagnostic methods with high accuracy for tuberculous pleurisy are urgently needed. In the present study, we evaluated the diagnostic accuracy of Xpert MTB/RIF, LAMP and SAT-TB assay with pleural fluids from culture-positive TBP patients.

**Methods:**

We prospectively enrolled 300 patients with exudative pleural effusions used as the samples for Xpert MTB/RIF, LAMP and SAT-TB assay. Of these, 265 including 223 patients diagnosed with TBP and 42 non-TBP patients used as controls were analyzed.

**Results:**

The sensitivities of Xpert MTB/RIF (27.4%), LAMP (26.5%) and SAT-TB assay (32.3%) were significantly higher than that of pleural effusion smear (14.3%, *X*^2^ = 20.65, *P* <  0.001), whereas they were much lower than expected for the analysis of pleural effusion samples. Both SAT-TB assay and Xpert MTB/RIF demonstrated high specificities (100%) and PPVs (100%), but the NPVs of all of the tests were < 22%. The area under ROC curve of pleural effusion smear, LAMP, Xpert MTB/RIF and SAT-TB assays was 0.524 (95% CI 0.431–0.617), 0.632 (95% CI 0.553–0.71), 0.637 (95% CI 0.56–0.714) and 0.673 (95% CI 0.6–0.745). SAT-TB assays had the highest AUC.

**Conclusion:**

Nucleic acid amplification tests are not the first choice in the diagnosis of tuberculous pleurisy. In this type of test, SAT-TB is recommended because of its low cost, relatively more accurate compared with the other two tests. This prospective study was approved by The Ethics Committee of the Shanghai Pulmonary Hospital (approval number: K19–148).

**Trial registration:**

ClinicalTrials.gov identifier: ChiCTR1900026234 (Retrospectively registered). The registration date is September 28, 2019.

## Background

Tuberculosis (TB), the leading cause from a single infectious agent, typically affects the lungs (pulmonary TB), but can also affect other sites (extrapulmonary TB). Extrapulmonary TB (EPTB) represented 14% of the 6.4 million incident cases notified in 2017, globally [1]. The most common form of EPTB is tuberculous pleurisy (TBP) [2]. However, the sensitivity of acid-fast bacilli (AFB) in pleural effusion (PE) smear is unacceptably low and non-*tuberculos Mycobacterium* (NTM) is also positive [3]. The definite diagnosis of TBP is made by detecting *Mycobacterium tuberculosis* (MTB) from PE or pleural tissue [4], but culturing M. tuberculosis will take 2–8 weeks to obtain the results, which can delay effective medical interventions [5]. Delayed antituberculosis treatment may result in pleural thickening or tuberculous empyema that requires surgical resolution [6, 7]. Therefore, diagnosis of TBP is sometimes referred to pleural biopsy. However, pleural biopsy is invasive and adds considerable cost to the workup. In addition, biopsy of pleural tissue for histological examination may still have false negative rate of about 20% [8]. Technological advances in nucleic acid amplification tests (NAATs) have led to breakthroughs in TB diagnosis with turnaround time under 2 h [9]. Xpert MTB/RIF (Xpert), endorsed by the Scientific and Technical Advisory Board of the WHO, integrates hemi-nested real-time *Mycobacterium tuberculosis*-specific DNA amplification and simultaneous detection of mutations in the rifampicin resistance-associated rpoB mutations [10]. However, the requirement of expensive specialized equipment and the high cost of the assay make it unaffordable for large-scale use in developing countries. Loop-mediated isothermal amplification (LAMP) is a DNA amplification at a constant temperature by one type of enzyme with rapid and simple features which make it a promising diagnostic method for point-of-care testing and for resources limited countries [11]. Simultaneous amplification and testing for detection of *Mycobacterium tuberculosis* complex (MTBC) (SAT-TB assay) is a relatively new method based on real-time fluorescence simultaneous isothermal RNA amplification. Since RNA is much more unstable than DNA, so SAT-TB assay (SAT-TB) has the advantage of lower false-positive rates and good reproducibility [12]. Previous studies of NAATs have demonstrated superior sensitivity and specificity for the diagnosis of pulmonary TB with sputum specimens [13–18]. However, there is still limited data on the performance of NAATs on the diagnosis of TBP with pleural fluid specimens. Whether these tests are sensitive enough to rule out TBP remains unclear.

Thus, we designed the current prospective study to evaluate the diagnostic performance of Xpert, LAMP and SAT-TB with PE specimens from confirmed TBP patients in a country with high TB incidence.

## Methods

### Patients

In this study, we prospectively screened all new patients with exudative pleural effusions who had been admitted to Shanghai Pulmonary Hospital for suspected active TBP from January 2017 to December 2018. Data regarding age, sex, history of anti-TB treatment, current symptoms, course of the disease, and comorbidities were obtained from each enrolled patient using a standardized questionnaire. The exclusion criteria for enrollment were as follows: < 18 years of age, seropositive for human immunodeficiency virus (HIV), and inability to provide PE for examinations. In this study the definite diagnosis of TBP is made by detecting *Mycobacterium tuberculosis* from the PE with BACTEC MGIT 960 culture. The patients with PE due to causes other than TB were used as controls. Enrolled patients for whom a definite diagnosis could not be made were excluded from our further analysis.

All of the patients had provided written informed consent for a protocol approved by The Ethics Committee of Shanghai Pulmonary Hospital (approval number: K19–148). Our study was performed in accordance with the Declaration of Helsinki with regard to ethical principles for research involving human subjects.

### Examinations

Each patient underwent physical examination, chest computed tomography (CT), blood T-SPOT.TB interferon-gamma release assay (T-SPOT.TB) and thoracentesis guided by ultrasound or CT. At least 40 mL of PE samples was collected from each patient during thoracentesis using a sterile syringe. Aliquots of each sample were simultaneously submitted for adenosine deaminase assay (ADA), lymphocyte percentage of total cells, cytology for malignant cells, bacterial culture and fungal culture, smear fluorescence microscopy (FM), BACTEC MGIT 960 culture (MGIT 960), Xpert, LAMP and SAT-TB immediately after collected from the patients. Phenotypic drug susceptibility testing (DST) to first-line drugs was performed by automatic MGIT 960. ADA was analyzed using a colorimetric assay (Diazyme Laboratories, Poway, CA, USA). T-SPOT.TB was performed as previously described [19]. BACTEC MGIT 960 (Becton Dickinson Life Sciences, Franklin Lakes, NJ, USA) was performed according to the standard procedure of the manufacturer [20]. SAT-TB was carried out using the method of AmpSure assay (Shanghai Rendu Biotechnology, Shanghai China) following the instructions of the manufacturer [18]. LAMP reactions were conducted with Loopamp DNA amplification kit (both from Eiken Chemical, Tochigi, Japan), as previously described [11]. Xpert (Cepheid, Sunnyvale, CA, USA) were performed according to the manufacturer’s instructions using a four-module GeneXpert machine and the results can be automatically generated by the machine. All tests were conducted at the TB reference laboratory in Shanghai Pulmonary Hospital by qualified technicians using routine quality control procedures. Since these tests are automatic, there is no need of blinding.

### Statistical analysis

Data was analyzed using Statistics for Windows (Version 18.0, Chicago, US: SPSS Inc.). Numerical variables were reported as mean ± standard deviation, and categorical variables were shown as number and percentage of observations. Diagnostic performance was assessed using sensitivity, specificity, positive predictive value (PPV), negative predictive value (NPV) and accuracy. Continuous variables were compared with t-test, while the comparison of categorical variables were made by Fisher’s exact test or Pearson’s chi-squared analysis, as appropriate. Differences were considered statistically significant when *P* -value ≤0.05. Receiver operating characteristic (ROC) curve analysis was performed to determine the power of these tests to distinguish TBP patients from non-TBP patients.

## Results

### Baseline demographic and clinical characteristics

We prospectively enrolled 300 patients. Thirty-five patients for whom a clear diagnosis could not be determined were excluded from further analysis. Finally, the remaining 265 were analyzed, including 223 patients diagnosed with TBP and 42 patients with pleural effusion due to causes other than TBP used as controls. Diagnosis in the non-TBP group included lung cancer (*n* = 12), bacterial pleurisy (*n* = 22), systemic lupus erythematosus (*n* = 1), and NTM infection (*n* = 7). The baseline demographic and clinical characteristics of 265 patients were summarized in Table 1. TBP patients were significantly younger (41.3 ± 17.6) than non-TBP patients (56.4 ± 13.9; *p* <  0.001), and had a longer course of disease (*p* <  0.001). However, non-TBP patients were more likely to have no intrapulmonary lesions (*p* <  0.001).

**Table 1:** Baseline demographic and clinical characteristics of 265 patients

	TBP (223 cases)	Non-TBP (42 cases)	*P*-value
Age, mean (SD) [range], years	41.3 (17.6) [15–83]	56.4 (13.9) [25–81]	< 0.001
Male gender, No. (%)	138 (61.9)	25 (59.5)	0.713
BMI^a^, median (range)	19.5 (15–27)	19.3 (14–29)	0.066
Fever (%)	129 (57.9)	22(52.4)	0.512
Course of disease [range], weeks1	5.2 [1–52]	2.1 [1–208]	< 0.001
Diabetes mellitus (%)	22 (9.9)	4 (9.5)	0.946
History of anti-TB treatment	19 (8.5%)	1 (2.4%)	0.167
ADA (U/liter) > 25	201 (90.13%)	10 (23.8%)	< 0.001
LP^b^ > 50%	118 (52.9%)	16 (38.1%)	0.078
Without intrapulmonary lesions	5 (2.2%)	12 (28.6%)	< 0.001
T-SPOT.TB on PBMCs^c^	208 (93.3%)	5 (11.9%)	< 0.001

^a^BMI, Body mass index; ^b^ LP, lymphocyte proportion; ^c^ PBMCs, Peripheral blood mononuclear cells

### Results of LAMP, Xpert MTB/RIF and SAT-TB assay

Table 2 summarized the results of various diagnostics tests. The sensitivities of Xpert (27.4%), LAMP (26.5%) and SAT-TB assay (32.3%) were significantly higher than that of PE smear (14.3%, *X*^2^ = 20.65, *P* <  0.001), whereas they were much lower than expected for the analysis of PE samples. As shown in Table 3, both SAT-TB assay and Xpert demonstrated high specificities (100%) and PPVs (100%), but the NPVs of all of the tests were < 22%. The accuracies of these tests were also far from satisfactory. In the non-TBP group, 4 patients with false-positive smear results were identified as NTM and 1 patient with bacterial pleurisy presented false-positive LAMP result. These results suggested that NAATs are suboptimal for the detection of M. tuberculosis in PE. To further explore the correlation between ADA level and these experimental detection rates, patients were divided into three groups according to ADA level: a low-ADA (< 40 IU/L) group (*n* = 64), a medium-ADA (40–70 IU/L) group (*n* = 117) and a high-ADA (> 70 IU/L) group (*n* = 42) (Table 4). There were no significant differences in the positive rate of these tests in different ADA level groups.

**Table 2:** Results of the diagnostic tests

Method	Diagnostic rate	
	TP (223 cases)	Non-TBP (42 cases)
PE smear	32 (14.3%)	4 (9.5%)
LAMP positive	59 (26.5%)	1 (2.4%)
Xpert positive	61 (27.4%)	0
SAT-TB positive	72 (32.3%)	0

*PE* Pleural effusion, *TBP* Tuberculous pleurisy

**Table 3:** Comparison of PE smear, SAT-TB, Xpert and LAMP result

Method	Sensitivity	Specificity	PPV	NPV	Accuracy
PE smear	14.3%	90.9%	88.9%	16.6%	26.4%
LAMP	26.5%	97.6%	98.3%	20.0%	37.7%
Xpert	27.4%	100%	100%	20.6%	38.0%
SAT-TB	32.3%	100%	100%	21.8%	43.0%

*PE* Pleural effusion

**Table 4:** Comparison of PE smear, SAT-TB, Xpert and LAMP results of each ADA level group

Method	PE smear	Xpert	LAMP	SAT-TB	*P*-value
Low-ADA(64)	7 (10.94%)	13 (20.31%)	16 (25.00%)	17 (26.56%)	0.123
Medium-ADA(117)	17 (14.53%)	32 (27.35%)	25 (21.37%)	34 (29.06%)	0.035
High-ADA(42)	8 (19.05%)	16 (38.10%)	18 (42.86%)	21 (50.00%)	0.024
Total	32	61	59	72	

Low-ADA group: ADA < 40 IU/L, Medium-ADA group: ADA =40–70 IU/L, High-ADA group: ADA > 70 IU/L

### Establishment of ROC curve

**Fig. 1:**
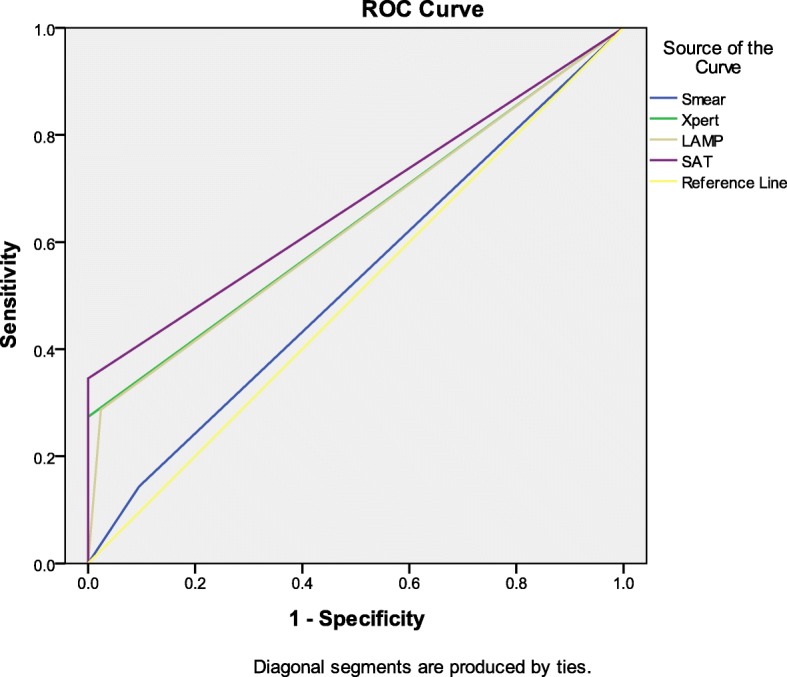
ROC curve of smears, LAMP, Xpert and SAT-TB

The area under ROC curve (AUC) of smears, LAMP, Xpert and SAT-TB was 0.524 (95% CI 0.431–0.617), 0.632 (95% CI 0.553–0.71), 0.637 (95% CI 0.56–0.714) and 0.673 (95% CI 0.6–0.745) (Fig. 1). SAT-TB had the highest AUC.

### Results of DST to first-line drugs

The result of phenotypic DST indicated that 20 patients (9.0%) were multidrug resistant tuberculosis (MDR-TB) and 1 patient (0.4%) was rifampicin resistant tuberculosis (RR-TB). The MDR/RR-TB rate was essentially higher in previously treated TBP (52.6%) than in primary TBP (5.4%, *X*^2^ = 45.47, *P* <  0.001). Xpert correctly identified 71.4% (15/21) of MDR/RR-TB cases (Table 5).

**Table 5:** Results of drug susceptibility testing

Susceptibility test	Primary TBP (*N* = 204)	Retreatment TBP (*N* = 19)	Total (*N* = 223)
MDR	10 (4.9%)	10 (52.6%)	20 (9.0%)
Rifampicin	1 (0.5%)	0	1 (0.4%)
PDR	9 (4.4%)	1 (5.3%)	10 (4.5%)
Isoniazid	6 (2.9%)	3 (15.8%)	9 (4.0%)
Ethambutol	1 (0.5%)	0	1 (0.4%)
Streptomycin	59 (26.5%)	1 (2.4%)	60 (26.9%)

*TBP* Tuberculous pleurisy, *MDR* Multidrug resistant, *PDR* Polydrug resistant

## Discussion

In this study, we evaluated LAMP, Xpert and SAT-TB assays for the diagnosis of TBP in PE culture positive patients, compared with PE smear and found that all these methods were suboptimal for the detection of MTB in PE, whereas each of them demonstrated high specificity. Similar previous investigations of NAATs for detecting MTB in PE have also reported modest sensitivities [21–24]. Touré et al. reported Xpert MTB/RIF with pleural liquid was positive in only 3.3% of 301 TBP patients [25]. Tyagi et al. conducted a meta-analysis, collecting 58 studies on pleural fluid-based Xpert MTB/RIF and found that the pooled sensitivity was inadequate [26]. IS1081- based LAMP was developed in a study by B. Yang et al., for the detection of MTB in PE, that was positive in 25% TBP patients (18 / 72), while no positive reaction was observed in non-TBP patients [27]. In a meta-analysis of 40 studies of NAATs for TBP, PAI et al. reported that these tests had low sensitivities (43–77%), but high specificities (95%) [28]. The reasons for the low sensitivity of NAATs in PE specimens but high sensitivity in sputum samples are not clear. The presence of inhibitory substances in PE is not a satisfactory explanation, as studies have shown that some substances of potential inhibitors of nucleic acid detection, such as RNases, were similar in sputum and non-sputum specimens [29]. The paucity of MTB in PE may play some role, but the low sensitivity is more likely to be relevant to techniques of nucleic acid extraction. Therefore, the consistent high specificities of NAATs indicated their potential role in confirming TBP as ‘rule-in’ tests and were not useful in excluding the disease. Caution should be exercised when interpreting negative NAATs results in PE.

In addition, it’s worth mentioning that ADA remains the most widely used diagnostic PE marker as a screening tool for TBP in resource-limited settings where tuberculosis is endemic, since it has the advantage of cost-effectiveness, efficiency, noninvasiveness, and ease of operation [30, 31]. In our current study, 201out of 223 TBP patients (90.13%) had an ADA level over 25 U/l, while 10 out of 42 (23.8%) non-TBP also had an ADA level over 25 U/l. Nevertheless, apart from tuberculosis, high ADA levels in lymphocytic pleural effusions have also been reported in mesothelioma, lymphoma, rheumatoid immune system diseases and other infectious disease [3, 32, 33].

One possible shortcoming of this article was the number of cases is relatively small, because the diagnostic index we used is culture positive of MTB in PE, the “gold standard” for the diagnosis of tuberculosis.

## Conclusion

In conclusion, our research and previous work by other groups have suggested that NAATs are not the first choice in the diagnosis of TBP. If this type of test must be selected, the SAT-TB assay is recommended because of its low cost, relatively high sensitivity and high specificity compared with the other two tests. The diagnostic measure for TBP with high efficiency, low cost, rapid and convenient operation remains to be further studied.

## Data Availability

All data generated or analyzed during this study are included in this published article. The datasets used and/or analysed during the current study are available from the corresponding author on reasonable request.

## Abbreviations

ADA: Adenosine deaminase assay
AFB: Acid-fast bacilli
CT: Computed tomography
DST: Drug susceptibility testing
EPTB: Extrapulmonary tuberculosis
FM: Fluorescence microscopy
HIV: Human immunodeficiency virus
LAMP: Loop-mediated isothermal amplification
MDR: Multidrug-resistant
MTBC: *Mycobacterium tuberculosis* complex
NAATs: Nucleic acid amplification tests
NPV: Negative predictive value
NTM: Non- tuberculos mycobacteria
PE: Pleural effusion
PPV: Positive predictive value
PTB: Pulmonary tuberculosis
ROC: Receiver operating characteristic
RR-TB: Rifampicin resistant tuberculosis
SAT-TB assay: Simultaneous amplification and testing methods for detection of MTBC
TB: Tuberculosis
TBP: Tuberculous pleurisy
T-SPOT.TB: Interferon-gamma release assay
WHO: World Health Organization
